# Differential Development of the Chordae Tendineae and Anterior Leaflet of the Bovine Mitral Valve

**DOI:** 10.3390/jcdd11040106

**Published:** 2024-03-29

**Authors:** Meghan Martin, Chih-Ying Chen, Timothy McCowan, Sarah Wells

**Affiliations:** 1 School of Biomedical Engineering, Dalhousie University, Halifax, NS B3H 4R2, Canada; meghanmartin@dal.ca; 2 Medical Sciences Program, Faculties of Science and Medicine, Dalhousie University, Halifax, NS B3H 4R2, Canada; janecy.chen@mail.utoronto.ca (C.-Y.C.); tm914510@dal.ca (T.M.); 3 Department of Pharmacology and Toxicology, University of Toronto, Toronto, ON M5S 1A8, Canada; 4 Integrated Science Program, Faculty of Science, Dalhousie University, Halifax, NS B3H 4R2, Canada

**Keywords:** heart valves, extracellular matrix, remodeling, elastin, cardiac development, collagen, valvulogenesis

## Abstract

There is increasing evidence that some adult mitral valve pathologies may have developmental origins involving errors in cell signaling and protein deposition during valvulogenesis. While early and late gestational stages are well-documented in zebrafish, chicks, and small mammalian models, longitudinal studies in large mammals with a similar gestational period to humans are lacking. Further, the mechanism of chordae tendineae formation and multiplication remains unclear. The current study presents a comprehensive examination of mitral anterior leaflet and chordae tendineae development in a bovine model (a large mammal with the same gestational period as humans). Remarkably distinct from small mammals, bovine development displayed early branched chordae, with increasing attachments only until birth, while the anterior leaflet grew both during gestation and postnatally. Chordae also exhibited accelerated collagen deposition, maturation, and crimp development during gestation. These findings suggest that the bovine anterior leaflet and chordae tendineae possess unique processes of development despite being a continuous collagenous structure and could provide greater insight into human valve development.

## 1. Introduction

The increasing global prevalence of heart valve disease, propelled by an aging population and increased lifespan, is a growing health concern. Mitral valve pathologies, affecting approximately 24.2 million individuals worldwide and exhibiting an age-dependent increase in prevalence [1], not only compromise valvular function but also lead to left ventricular remodeling [2]. The current reliance on surgical interventions as the sole treatment for valve diseases further increases the strain on healthcare systems.

Several adult mitral valve pathologies have been linked to developmental defects during valvulogenesis [3,4,5]. For example, mutations in Dachsous cadherin-related 1 (*DCHS1*), the gene that codes for an atypical cadherin protein, have been identified in families with non-syndromic mitral valve prolapse [4,6,7]. Mutations in *DCSH1* in developing mice are associated with loss of actin organization, one of the key steps in the mechanotransduction pathway [3]. Mice from these models show thickened fetal leaflets with abnormal extracellular matrix (ECM) organization and eventually develop myxomatous mitral valve disease in adulthood [3,4]. These works show the clinical importance of understanding the processes and patterns of mitral valve formation throughout gestation. 

Fetal mitral valve development has been well-described in early and late gestational stages in many species, such as mice, rats, pigs, and chicks. Work on the single atrioventricular valve (AV) in developing zebrafish has also been used to evaluate valvulogenesis. In early gestation in humans [8], mice [9,10,11], chicks [12,13], and zebrafish [14,15,16], the formation of the endocardial cushions, comprised of a gelatinous extracellular matrix surrounded by endocardial cells, sets the foundation for valve development. Endocardial and epicardial cells undergo endothelial-to-mesenchymal cell transition (EMT) and migrate into the cushions, where they proliferate and differentiate into valvular interstitial cells (VICs) [8,16,17,18,19]. These cell types contribute differently to the different AV leaflets, where the anterior mitral valve leaflet is said to be derived from predominantly endocardial cells whereas the posterior leaflet is derived from epicardial cells [19]. The endocardial cushions protrude into the lumen and change blood flow from oscillatory to unidirectional pulsatile flow [20,21], increasing shear stress and activating mechanotransduction pathways [21]. The mechanotransduction pathways have been extensively documented in the literature [13,20,21,22], particularly in zebrafish models [14,23,24,25].

Ongoing increases in flow and tension during gestation direct further development and maturation of the mitral apparatus. By late gestation in humans [26], mice [27,28,29,30], chicks [20,21,22,30,31,32,33], and pigs [34,35,36], the endocardial cushions are remodeled into the more mature fibrous leaflet and associated chordae tendineae through cellular proliferation, condensation, and deposition of fibrous ECM proteins [3,27,28,29,37,38]. Increasing tension becomes the dominating force on the primordial valve, activating pathways of leaflet thinning and ECM remodeling [21]. Collagen fibers accumulate and become organized within the atrialis, fibrosa/ventricularis, and part of the spongiosa layer [27,28,33,34]. Fully formed elastic fibers are not found in the leaflets until the second trimester (within the atrialis) and then continue to increase over gestation in pigs [34] and humans [39,40]. Notably, in mice, the anterior mitral valve leaflet forms while attached to the underlying myocardium, delaminating around embryonic day 15 [29,41]. The delaminated leaflet maintains a papillary muscle connection through a single chordae “bridge” [27,30] that does not divide into multiple, branched chordae tendineae until late gestation [5,27]. Interestingly, rats do not develop multiple branched chordae until after birth [42]. 

Postnatally, the closure of fetal heart shunts elevates transvalvular pressures in the left side of the heart, resulting in significant changes to the anterior mitral valve leaflet structure, as observed in mice [3,27], pigs [34,35,36], and cattle [43]. These changes include increased thickness, collagen content, collagen crosslinking, and collagen alignment, accompanied by heightened cell remodeling activity, as indicated by elevated levels of alpha-smooth muscle actin, lysyl oxidase, and matrix metalloproteinases (MMP) 2 and 9 [34,35,36,37,43,44,45]. This leads to the characteristic trilaminar organization of the leaflet after birth [34,35,36] in the same porcine models. 

What is missing is our understanding of the mechanism and processes underlying mitral valve development in mid-gestation. While cellular activity is well-characterized over this interval [3,5,20,22], ECM deposition and organization remain poorly characterized. Also missing is our understanding of valvulogenesis in large animal models with longer gestational periods, providing an extended timeframe for valve development. While late gestation in the porcine model is well-characterized [34,35,36,37], a comprehensive understanding of histological changes and ECM-protein quantification over time in these larger mammals remains lacking. Moreover, the processes of formation and further development of branched chordae tendineae in large animal models have received limited attention in the literature, aside from brief mention in studies on pigs [34] and humans [26,46] that report the presence of branched chordae tendineae by late gestation. 

The objectives of this study were to characterize anatomical and histological changes in both the mitral valve anterior leaflet and chordae tendineae in a large animal model (bovine). Like humans, cattle have a similar four-chambered heart structure, nine-month gestational period, substantial size, and similar transvalvular pressures [47], thus serving as an excellent model for characterizing mitral valve valvulogenesis throughout gestation. Bovine tissues are also commonly used in studies on cardiovascular mechanics and in tissue engineering, where chemically-modified bovine pericardial tissues are used in the development of bioprostheses.

Remarkably distinct from small mammals, branched chordae tendineae were present within the first trimester, where attachments increased only until birth while the anterior leaflet grew both during gestation and postnatally. Bovine chordae further demonstrated accelerated collagen deposition, maturation, and crimp development during gestation. However, elastic fibers appeared in the leaflet ahead of the chordae suggesting that elastic fiber deposition and organization is the final step of chordae formation. These findings suggest that the bovine anterior leaflet and chordae tendineae possess unique, differential processes of development with distinct timelines despite them being a continuous collagenous structure.

## 2. Materials and Methods

### 2.1. Tissue Harvest and Anatomical Dimensions 

Protocols for the harvesting of bovine (*Bos taurus*) tissues were approved by the University Committee on Laboratory Animals at Dalhousie University. Hearts from both male and female fetal calves were obtained from two local abattoirs immediately following slaughter. Gestational age was determined from the fetal crown-to-rump length (CRL) using the following equation [48]:

$$Gestational\;age\;=\;8.4+0.087\left(CRL\right)+5.46{\left(CRL\right)}^{\frac{1}{2}}$$

Fetuses ranged in age from 60 to 270 days gestation (full term). The mitral valve anterior leaflet and associated chordae were excised, laid flat, and photographed with a reference scale bar. Images were imported into image analysis software (ImageJ, National Institutes of Health, Bethesda, MD, USA). The freehand selection tool was used to outline the leaflet’s perimeter to calculate the total leaflet area. The straight-line selection tool was used to measure the circumferential lengths, midline radial lengths, the distance between the two primary strut chordae (termed “strut distance”), and the position of the midline from the leaflet attachment site (termed “strut position”). Strut position and distances were normalized to leaflet radial and circumferential lengths, respectively. The multipoint tool was used to determine the number of chordal attachments to the anterior leaflet.

### 2.2. Collagen Biochemical Analysis

For biochemical assays, whole anterior mitral valves were placed in phosphate-buffered saline (PBS)–soaked gauze before being immediately stored in the −86 °C freezer until testing. Upon thawing, a radial segment of the anterior leaflet (taken from the free edge to the fixed edge between the strut chordae attachments) and a segment of the strut chordae (from the leaflet attachment to the papillary muscle, no branches included) were excised, and wet weights of the samples were recorded in milligrams. Wet weights of the samples varied based on gestational age, but the chordae ranged from approximately 1–15 mg and for the leaflet, 5–40 mg. First trimester valves were too small to yield samples for biochemical assay, so this analysis includes only samples aged 106 days to 270 days (full term).

To measure the levels of acid and pepsin-soluble collagen, the Sircol™ Soluble Collagen assay (Biocolour, Carrickfergus, UK) was used according to the manufacturer’s protocols. This form of collagen can be interpreted as newly synthesized collagen not sufficiently crosslinked into the network to resist solubilization. To isolate the soluble collagen, tissue samples were placed in 0.1 mg/mL pepsin (Sigma-Aldrich, Oakville, ON, Canada) in 0.5 M acetic acid overnight at 4 °C and the supernatant was used for the Sircol™ Soluble Collagen assay. The remaining tissue residues underwent the Sircol™ Insoluble Collagen assay (Biocolour, Carrickfergus, UK) following the manufacturer’s protocol. Insoluble collagen is interpreted as mature crosslinked collagen. The residues were denatured at 65 °C for three hours in acetic acid, causing all tissue to breakdown and crosslinked collagen to release into solution.

All samples were assayed in duplicate and compared to a set of blanks (water) and known concentration of standards comprised of bovine collagen. Following each assay, a microplate reader (Varioskan LUX; Thermo Scientific, Waltham, MA, USA) was used to measure the dye absorbance of each prepared plate at a wavelength of 555 nm. Collagen content was calculated from the sample absorbance and normalized against wet tissue weight to get collagen concentration in µg/mg wet tissue weight.

### 2.3. Histology

One-half of the anterior leaflet with associated chordae tendineae was cut from fetal samples in the second and third trimesters (91–270 days gestation). For first trimester samples (0–90 days gestation), the whole anterior leaflet and chordae were used due to their small size. Samples were fixed (10% buffered neutral formalin), embedded in paraffin, and sectioned into 4 µm serial transverse longitudinal sections. To examine collagen alignment and crimp, sections were stained with picrosirius red and hematoxylin. To visualize cells and the localization of ECM proteins, sections were stained with Russell-Movat Pentachrome Stain Kit (Statlab, TX, USA) according to the manufacturer’s instructions. This kit stains collagen yellow-orange, mature elastin dark purple, muscle tissue and blood cells red, glycosaminoglycans blue-green, and cell nuclei dark-red–purple. Movat-stained slides were imaged using a Pannoramic MIDI II and viewed with the Caseviewer software (3DHistech, Budapest, Hungary). 

### 2.4. Collagen Crimp Analysis 

**Figure 1:**
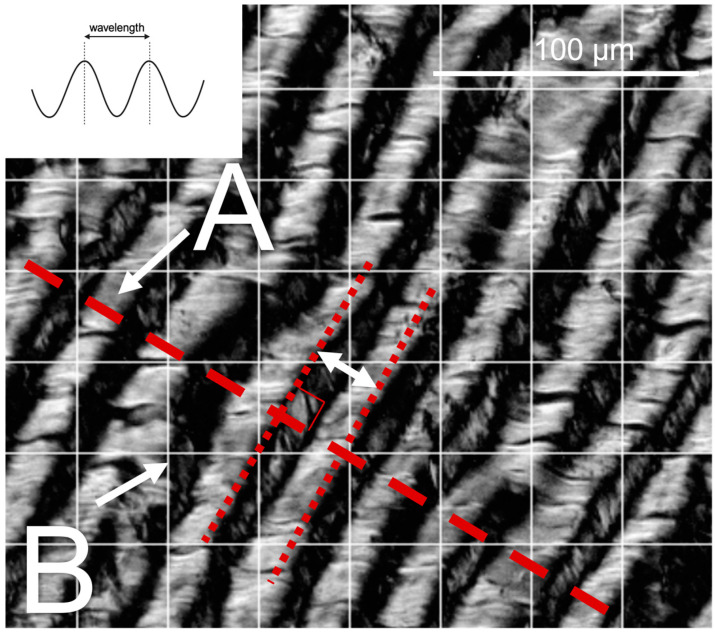
Methods used for collagen crimp measurement. The inset schematic depicts wavelength measured as the distance between adjacent “peaks” in the crimp waveform. Representative polarized light microscopy image shows crimp length measurements along a line normal to the crimp direction (A). Crimped area measurements were taken at each intersection of gridlines (B), as the ratio of crimped-to–total-grid-points (grid = 0.01 mm^2^/square) to calculate the area percentage of tissue occupied by crimp.

Images of picrosirius-red–stained sections were taken using a Nikon Eclipse E600 light microscope (Tokyo, Japan) (Nikon Instruments Inc., Melville, NY, USA) equipped with a polarizer and an AmScope 10MU1400 digital camera (AmScope, Irvine, CA, USA) to visualize collagen crimp. Collagen crimp is a macroscale organization of the collagen fibers that leads to a “waviness” (Figure 1). Functionally, this crimp is important for tensile deformation where this crimp is first flattened upon tensile loading, leading to the toe region of the typical collagen stress-strain curve before the collagen fibers are engaged [49]. Images were taken at the X40 objective along the circumferential direction of the leaflet and radial length of the chordae. Collagen crimp was characterized (as described previously [50,51,52], with minor modifications) by (a) crimp wavelength (peak-to-peak distance of one crimp period, Figure 1) and (b) the percentage of tissue area that was crimped. Briefly, crimp wavelength was analyzed in ImageJ where a 4-quadrant grid (0.01 mm^2^/square) was placed over the image, and two areas per grid were measured. A straight line in the middle of the fiber was drawn and all the crimp peaks parallel to the line were measured over that distance (Figure 1). The crimp wavelength measurements were averaged across all grids to give one value per image. Crimp area was measured by placing a 600 µm^2^/square grid overtop the same images (Figure 1) and grid points were counted as (i) in contact with crimped tissue, (ii) uncrimped tissue, and (iii) empty space (which was then omitted from total grid count). The ratio of crimped grid points to the total grid count was used to calculate the percentage of crimped tissue area.

### 2.5. Statistical Analysis

To assess the changes in each parameter during fetal development, data were plotted as a function of gestational age in days and fitted with a linear least-squares regression. In the case of leaflet area, a power relationship provided the best fit, and the *p* value was assessed from a linear regression log transformation. Each data point represents *n* = 1 animal. The regression was considered significant when *p* < 0.05. To compare the rate of change within any single parameter during fetal development between tissues (chordae versus leaflet), the regression slopes were compared using an analysis of covariance (ANCOVA). Differences between the slopes were considered significant when *p* < 0.05. For the soluble collagen data, there was no significant change with gestational age in chordae or leaflet so comparisons between those tissues were made using the gestational averages using an unpaired two-sample *t*-test. All statistical analyses were conducted using R [53]: specifically, the car package [54], mvnormtest package [55], DescTools package [56], and devtools package [57].

## 3. Results

### 3.1. Chordae Division Occurs Only during Gestation Whereas Leaflet Area Continues to Increase Postnatally

**Figure 2:**
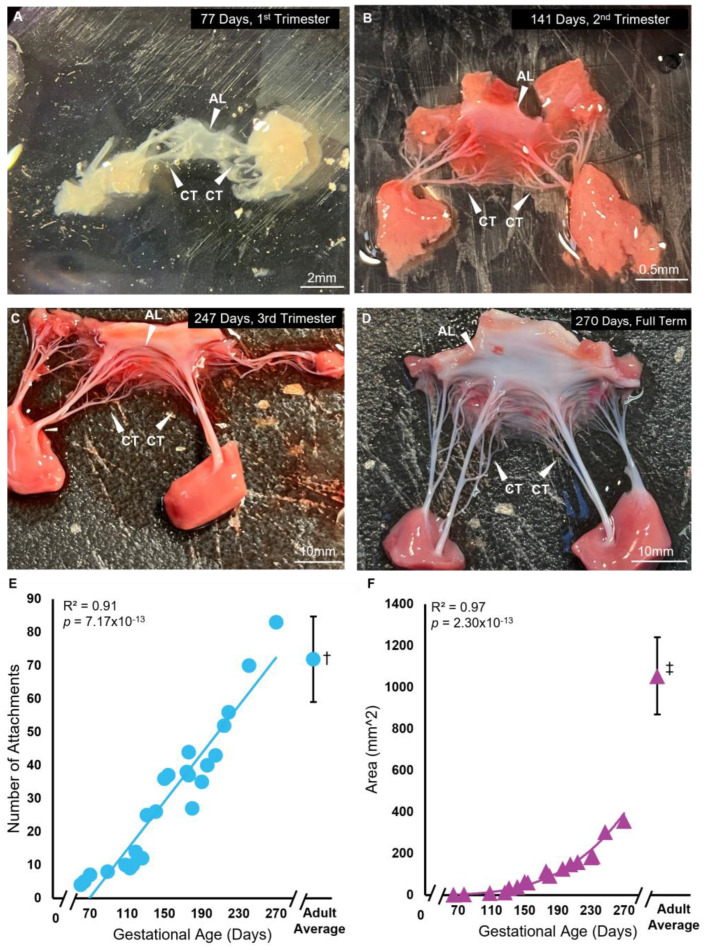
Branched chordae are present within the first trimester of bovine mitral valve development, with chordae number and leaflet area increasing throughout gestation. (**A**–**D**) Representative images of bovine fetal mitral valve anterior leaflets and chordae tendineae from first trimester (77 days gestation), second trimester (141 days gestation), third trimester (247 days gestation), and full-term (270 days). White arrows denote the anterior leaflet (AL) and chordae tendineae (CT). (**E**) Number of chordae tendineae attachments plotted as a function of gestational age, showing a significant correlation between these variables. Each data point represents *n* = 1 animal. † The average value (±SD, *n* = 13) from adult animals taken from Scott 2016 [58] is shown for comparison to fetal data. (**F**) Anterior leaflet area in mm^2^ plotted as a function of gestational age in days, showing a significant power relationship between these variables. ‡ The average value (±SD, *n* = 11) from adult animals, taken from Wells et al., 2012 [59] (leaflet area) for comparison to fetal data.

The anterior mitral valve leaflet underwent significant increases in the area and number of chordae tendineae attachments during gestation (Figure 2). The anterior leaflet area demonstrated a non-linear power-relationship increase over gestation from 64 days (2.25 mm^2^) to 270 days (357.78 mm^2^), yet remained approximately 3 times lower than the adult average (1054.46 ± 185 mm^2^) (Figure 2A–D,F). The adult averages given here are from our previously published data using heifers (never-pregnant, sexually mature female cattle) [58,59]. Chordae attachments also increased linearly during gestation from 60 days (4 attachments) to 270 days (83 attachments) (Figure 2E). However, unlike leaflet area, the number of chordae attachments reached adult-like numbers (72 ± 13) by full term. 

**Figure 3:**
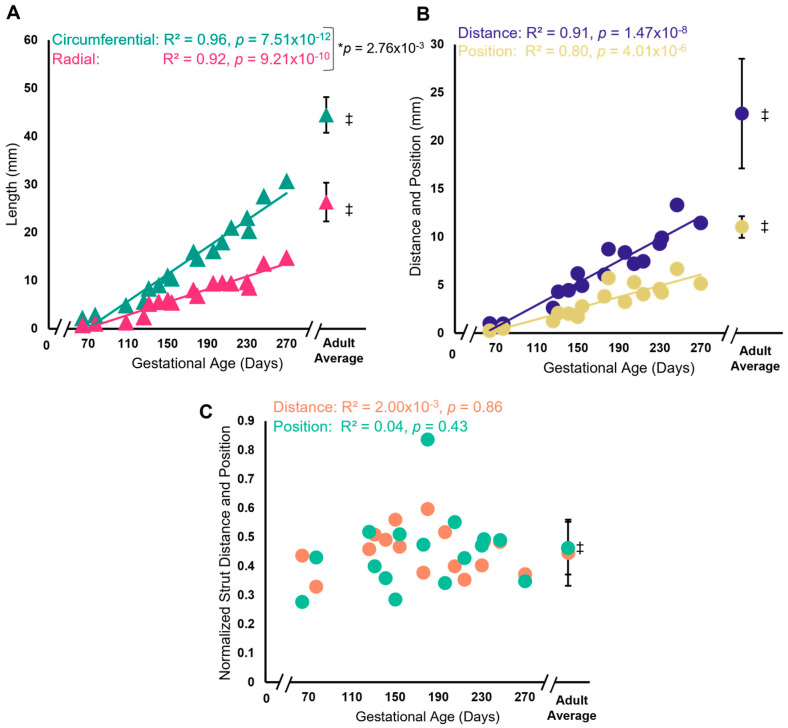
Despite radial and circumferential expansion of the leaflet over gestation, the normalized strut chordae distance and position remained the same over gestation. (**A**) Circumferential length of the anterior leaflet (green triangles) and radial length (pink triangles) in mm plotted as a function of gestational age, showing a significant correlation between these variables. * Denotes a significant difference in slope values (*p* = 0.04) analyzed via analysis of covariance. (**B**) Distance between the strut chordae (blue circles) and strut chordae position (yellow circles) from the fixed edge of the leaflet in mm plotted as a function of gestational age, showing a significant correlation between these variables. (**C**) Strut distance normalized to circumferential length (orange circles) and strut position normalized to radial length (green circles) plotted as a function of gestational age, showing no change with gestational age. Each data point represents *n* = 1 animal. ‡ The average values (±SD, *n* = 11) from adult animals, taken from Wells et al., 2012 [59] are shown as green triangles (circumferential length), pink triangles (radial length), blue circles (strut distance), yellow circles (strut position), green circles (normalized strut position) and orange circles (normalized strut distance) for comparison to fetal data. Note the axis breaks on all four graphs.

Interestingly, the normalized relationship between strut chordae attachment distance and position did not change throughout gestation despite increasing leaflet size (Figure 3). Both the circumferential and radial lengths increased during gestation, with a greater rate of increase in the circumferential direction (Figure 3A). As with area, fetal leaflet lengths at full term remained lower than the adult average (from the same previously published data [59]). Similarly, the distance between strut chordae attachments and strut position from the fixed edge increased over gestation but remained lower than the adult average at full term (Figure 3B). However, when normalizing strut distance (to circumferential length) and position (to radial length), the relationship remains the same throughout gestation (Figure 3C). Further, this relationship does not appear to change postnatally (Figure 3C). 

### 3.2. In Leaflet and Chordae, Mature Collagen Content Increases over Gestation despite Unchanged Levels of Newly Synthesized Collagen

**Figure 4:**
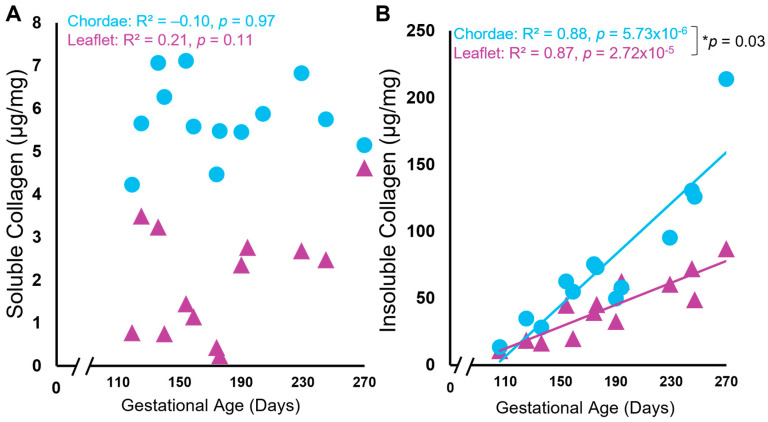
Levels of soluble (newly synthesized) collagen remained unchanged during gestation while insoluble (mature) collagen content increased in both the anterior leaflet and chordae tendineae. Concentration of soluble (**A**) and insoluble (**B**) collagen (in µg/mg wet weight) of the anterior leaflet (purple triangles) and strut chordae tendineae (blue circles) as a function of gestational age. Each data point represents *n* = 1 animal. Note the break in the x-axis both graphs. Significant regression lines are shown as solid lines and (*p* < 0.05). * Denotes significant difference in slope (*p* = 0.03) values analyzed via analysis of covariance.

Biochemical analysis of both soluble and insoluble collagen showed unexpected patterns (i) during gestation and (ii) between the leaflet and chordae. Soluble “newly synthesized” collagen concentration was surprisingly unchanged over gestation in both tissues, with consistently higher levels in the chordae (averages over gestation in chordae = 5.76 + 0.89 µg/mg vs. leaflet = 2.03 + 1.34 µg/mg, *p* = 1.51 × 10^−8^, Figure 4A). By contrast, the concentration of insoluble “mature” collagen increased linearly over gestation in both the leaflet and chordae, but at a faster rate in the chordae (Figure 4B).

### 3.3. Collagen Crimp Develops Earlier in the Chordae versus the Leaflet

**Figure 5:**
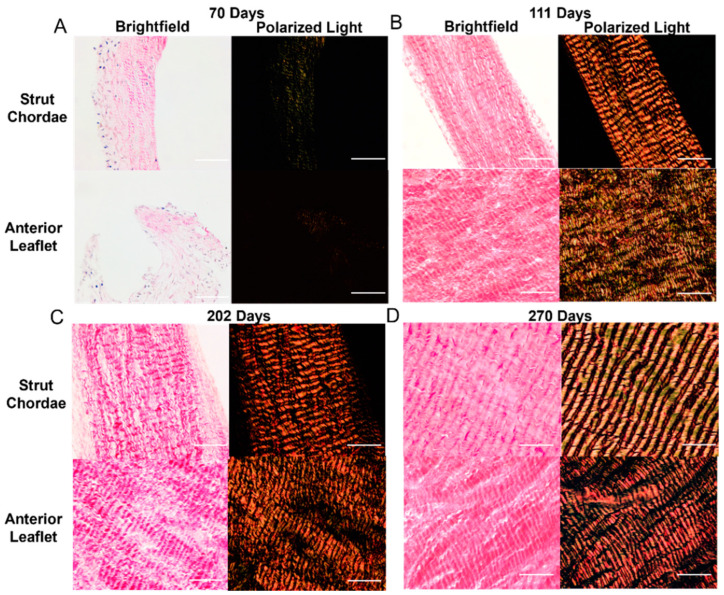
Representative images of collagen crimp throughout gestation in both the anterior leaflet and chordae tendineae. (**A**–**D**) Brightfield and polarized light images of the mitral anterior leaflet and strut chordae from first trimester (70 days gestation), second trimester (111 days gestation), third trimester (202 days gestation), and full-term valve (270 days gestation). Images taken at 400×. Scale bar, 0.05 mm. Measurements of collagen crimp wavelength (distance between adjacent wave “peaks”) with gestational age are shown in Figure 6B.

**Figure 6:**
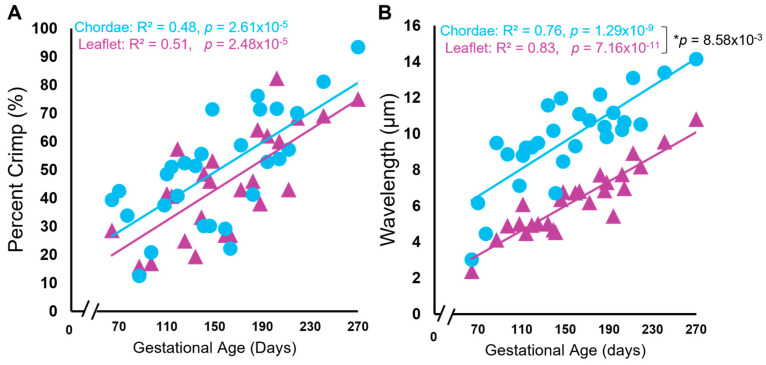
Collagen crimp is established, and collagen crimp wavelength increased throughout gestation in both the anterior leaflet and chordae tendineae. Percent area of crimped tissue (**A**) and collagen crimp wavelength in µm (**B**) of the anterior leaflet (purple triangles) and strut chordae tendineae (blue circles) as a function of gestational age. Each data point represents *n* = 1 animal. Note the break in the x-axis for both graphs. Significant regression lines are shown as solid lines and (*p* < 0.05). * Denotes significant difference in slope (*p* < 0.05) values analyzed via analysis of covariance.

There were also changes to collagen crimp in the leaflet and chordae during gestation, with the chordae leading the leaflet in crimp development. Figure 3 shows representative bright-field and polarized-light images of the picrosirius-red–stained leaflet and chordae from fetuses at 70 days gestation (Figure 5A), 111 days gestation (Figure 5B), 202 days gestation (Figure 5C), and 270 days gestation (full term) (Figure 5D) where increasing crimp wavelength over gestation is visualized in both tissues. Measurements of crimp wavelength are shown in Figure 6B. 

Leaflet and chordae showed identical linear increases in the percent of crimped collagen over gestation (Figure 6A). Collagen crimp wavelength also increased linearly during gestation in both the leaflet and chordae but with a slightly higher rate of increase in the chordae (Figure 6B).

### 3.4. Bovine Mitral Chordae and Leaflet Collagen Fibers Are Laid Down in Their Adult-like Orientations

**Figure 7:**
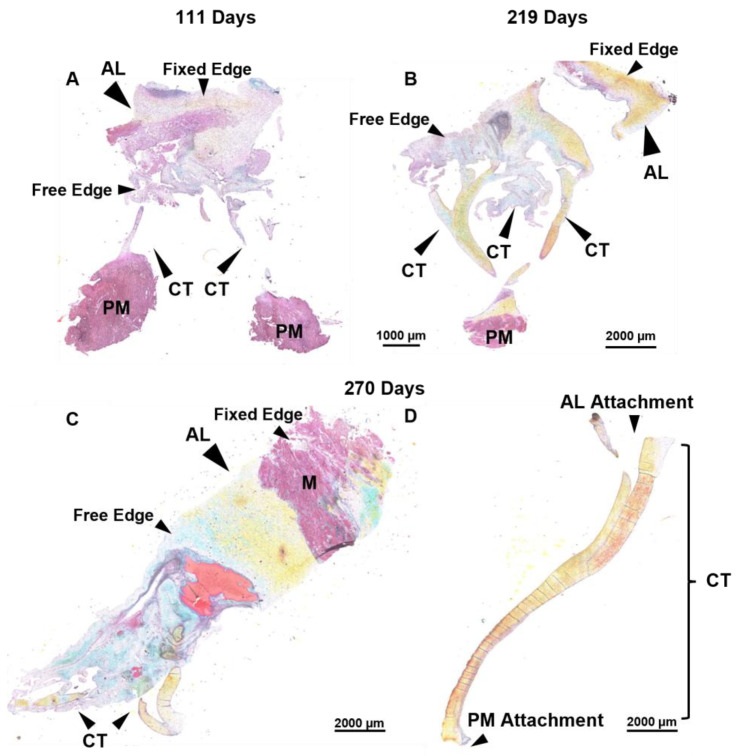
Throughout the second trimester to full term, the anterior leaflet and chordae tendineae are comprised predominantly of collagen. (**A**–**D**) Representative images of extracellular matrix staining (Movat Pentachrome) from early second trimester (111 days gestation), third trimester (219 days gestation), and full term (270 days). Black arrows denote the anterior leaflet (AL) and chordae tendineae (CT). Papillary muscle (PM) and myocardium (M) are labeled. Note that these are transverse longitudinal sections, therefore the fixed edge and the free edge are labeled for orientation. For the strut chordae in (**D**), anterior leaflet attachment and papillary muscle attachment are labelled for orientation. Collagen is stained yellow-orange, elastic fibers dark purple, muscle tissue and blood cells red, glycosaminoglycans blue-green, and cell nuclei dark red-purple. Scale bar varies per image.

Movat pentachrome staining was used to visualize ECM composition and organization of the mitral valve anterior leaflet and chordae tendineae (Figure 7). To capture the whole anterior mitral apparatus, single planar longitudinal sections of the whole valve were taken, as seen in the representative images below. This staining showed that the fetal leaflet and chordae were mostly comprised of glycosaminoglycans and collagen, with some infiltration of myocardium present in the leaflets (Figure 7A,C). 

**Figure 8:**
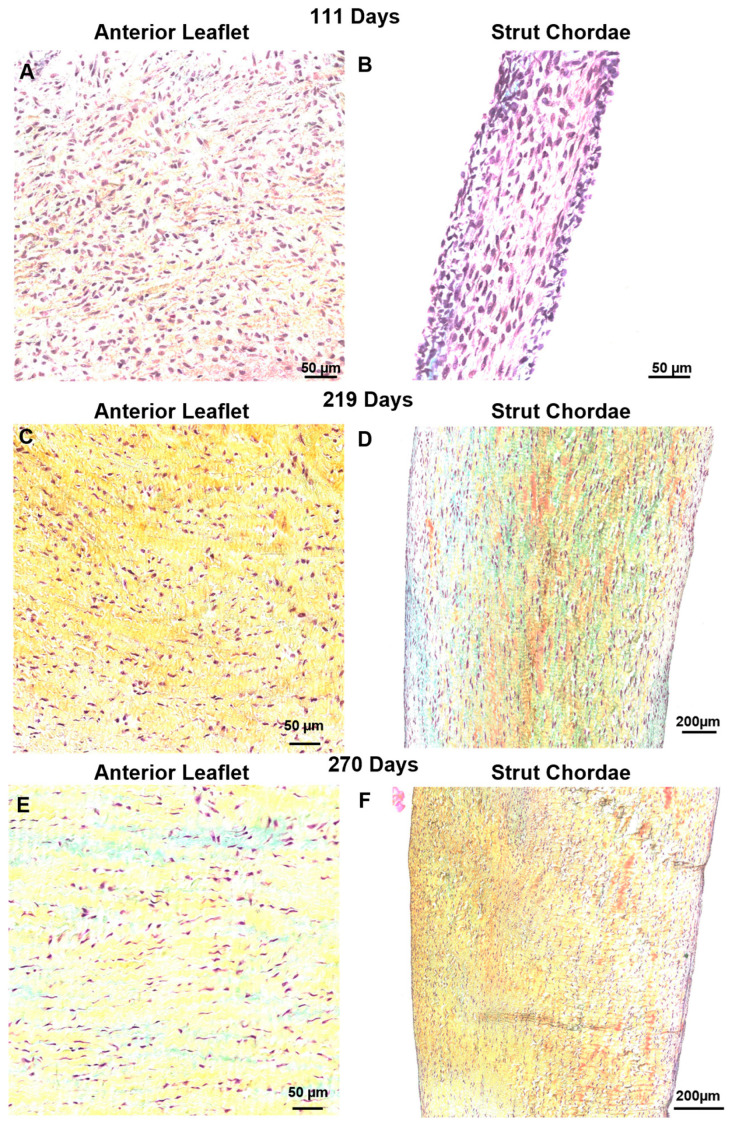
Throughout gestation, there were no major changes to collagen (stained yellow-orange) fiber orientation in the anterior leaflet or chordae tendineae. Circumferential sections of the leaflet showed primarily circumferentially-aligned collagen fibers and the longitudinal sections of the strut chordae showed primarily longitudinally-aligned collagen fibers. Magnified sections of representative images of Movat-Pentachrome–stained mitral anterior leaflets (**A**,**C**,**E**) and strut chordae tendineae (**B**,**D**,**F**). (**A**,**B**) Early second trimester (111 days gestation). (**C**,**D**) Third trimester (219 days gestation). (**E**,**F**) Full term (270 days). Collagen is stained yellow-orange, glycosaminoglycans blue-green, and cell nuclei dark red-purple. Scale bar varies per image.

Surprisingly no major changes to collagen fiber orientation were revealed in either the leaflet or chordae throughout gestation (Figure 8). The leaflet was comprised of collagen aligned circumferentially (Figure 8A,C,E) while the chordae were dominated by longitudinally-aligned collagen, as observed in mature animals (Figure 8B,D,F). Sparse staining of glycosaminoglycans and many cell nuclei were present in leaflet and chordae throughout gestation (Figure 7 and Figure 8).

### 3.5. Contrary to Collagen Patterns, Elastic Fibers Appear in the Leaflet Ahead of the Chordae

**Figure 9:**
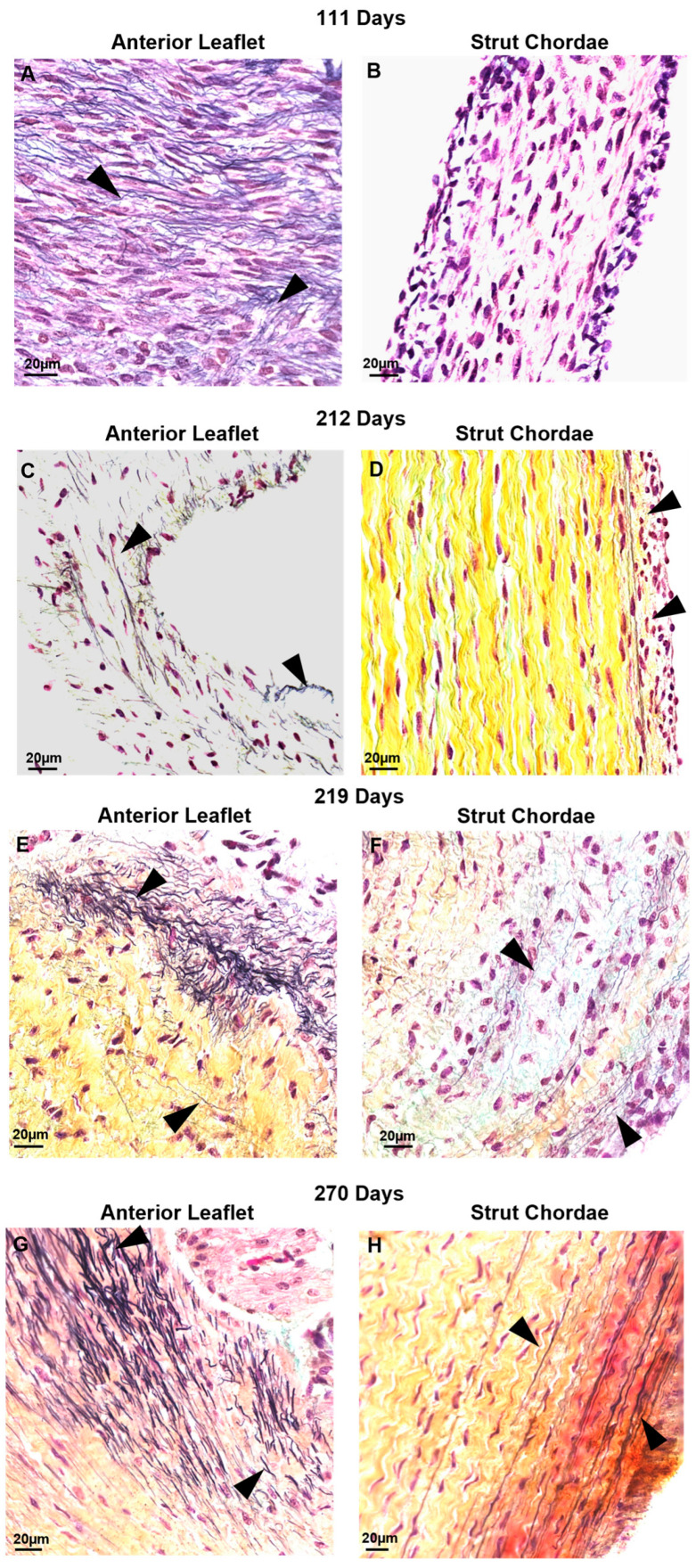
Elastic fibers (black arrows) appeared first in the anterior leaflet before being deposited within the chordae tendineae. Representative images demonstrating the presence of elastic fibers near the fixed edge in Movat-Pentachrome–stained mitral anterior leaflet (**A**,**C**,**E**,**G**) and strut chordae tendineae (**B**,**D**,**F**,**H**) throughout gestation. (**A**,**B**) Valve in early second trimester (111 days gestation), (**C**,**D**) third trimester (212 days gestation), (**E**,**F**) third trimester (219 days gestation), and (**G**,**H**) full term (270 days). Collagen is stained yellow-orange, elastic fibers dark purple, and cell nuclei dark red-purple. Elastic fibers first appeared in the leaflet in early second trimester (**A**) near the fixed edge. Elastic fibers accumulated throughout gestation, extending into the rest of the leaflet (**C**,**E**,**G**). Elastic fibers were not present in the chordae during second trimester (**B**), appearing only sparsely at the outer edges of the chordae in early third trimester (**D**) then increasing in density and length until full term (**F**,**H**). By late gestation, elastic fibers were also present within the bulk of the chordae (**H**).

There were also differential patterns of elastic fiber deposition in the leaflet and chordae over gestation. Elastic fibers first appeared in the leaflet during the early second trimester (111 days, Figure 9A) near the fixed edge. Elastic fibers accumulated through gestation, extending into the rest of the leaflet (Figure 9C,E,G). Distribution of elastic fibers in the leaflet was non-uniform throughout gestation, with some fields showing elastic fibers and others devoid of elastic fibers. By contrast, elastic fibers were not present in the chordae during the second trimester (Figure 9B), appearing only sparsely at the outer edges of the chordae in early third trimester (212 days, Figure 9D) with fibers increasing in density and length until full term (Figure 9F,H). By late gestation, elastic fibers were also present within the bulk of the chordae (Figure 9F,H).

## 4. Discussion

This study has described, for the first time, the structural changes in the mitral valve anterior leaflet and chordae tendineae during fetal development in a large animal model with a gestational period similar in length to that of humans. We found that—in striking contrast to small mammals—bovine mitral valve chordae tendineae rapidly develop over gestation, achieving their adult-like architecture by full term. This was accompanied by a rapid accumulation and maturation of ECM that outpaced that in the leaflet. Despite the continued expansion of leaflet area postpartum, the number of chordae attachments remained unchanged, suggesting that the mechanism driving chordae division is active only during fetal development. Thus, despite a nearly continuous collagen fiber architecture between the mitral valve anterior leaflet and chordae tendineae, their development appears to be more unique, each growing and maturing along independent timelines. These results provide an intriguing new window into the processes that could occur in human mitral valve development—where the timeline (in both fetal humans and bovines) for valve formation takes place across a much longer period than that in small mammals. Further, as mentioned above, bovine hearts are also four-chambered structures with similar transvalvular pressures to that of humans [42]. 

**Figure 10:**
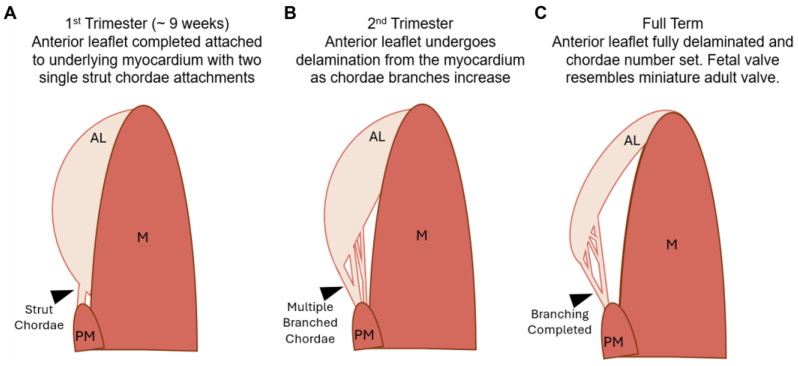
Schematic depicting the stages of development in the fetal bovine mitral valve anterior apparatus. (**A**) In the first trimester, the anterior leaflet (AL) is fully attached to the underlying myocardium (M). Two single strut chordae (denoted with black arrow), not attached to the myocardium, are attached to the leaflet and protrude from the papillary muscle (PM). (**B**) In the second trimester, the anterior leaflet delaminates from the underlying myocardium. Multiple branched chordae are now present. (**C**) By full term, the anterior leaflet has achieved it’s adult-like architecture. The leaflet has fully delaminated and chordae branching has ceased.

The main difference noted in fetal mitral valve formation between large and small mammals was in anatomical development. In mice, the anterior leaflet develops while remaining predominantly attached to the underlying myocardium [29,41]. Further, branched chordae tendineae are not formed until late gestation in mice [38] and postpartum in rats [41]. This has been attributed to the lower ventricular pressures during fetal development in small mammals, reducing the need for chordae during gestation [41]. While our bovine model demonstrated similar anterior leaflet attachment to the myocardium, this was only during the early first trimester and, contrary to mice, branched chordae were present at this time (Figure 2A and Figure 10A). The appearance of the first chordae in the bovine mitral valve (~9 weeks) was similar to what has been observed in humans, where a single distinct chorda was noted at approximately 12 weeks [26]. Branching and multiplication of chordae continued throughout gestation (Figure 2A–E and Figure 10B) until the adult-like architecture (i.e., number of attachments to the anterior leaflet) was reached by full term (Figure 10C). Therefore, by late gestation, the fetal bovine mitral valve resembles a miniature adult mitral valve, again similar to what has been observed in human mitral valve development [26]. If the emergence of chordae is indeed linked to ventricular pressure, a threshold of ventricular pressure (and local tissue tension) may be required for chordae formation. If that is the case, this threshold is reached in large mammals (such as humans and cattle) earlier during development (first trimester) compared to small mammals (late gestation). 

In the mitral valve of small mammals, collagen fibers are first laid down without a primary orientation before becoming more oriented in late gestation [28,33]. In our bovine model, collagen fibers were laid down in their adult orientation in the fetal anterior leaflet and chordae tendineae. At the fixed edge of the leaflet, collagen was predominantly oriented in the circumferential direction throughout gestation, as it is in adults [60]. Similarly, in the chordae collagen alignment was predominantly longitudinal throughout gestation, as it is in adults [61]. This further suggests that, in large mammals, the requirement for a functional mitral valve occurs earlier in gestation. 

Not surprisingly, the concentration of insoluble, mature collagen increased over gestation in both the leaflet and chordae. This change parallels increasing gene expression and presence of collagen seen in developing valves in other species, as demonstrated through RT-qPCR, picrosirius-red staining and immunohistochemistry [27,28,30,33,35,41,62,63,64]. Mature collagen accumulation in the chordae outpaced that in the leaflet, again reflecting the differential development of these tissues, with chordae tendineae undergoing rapid development during gestation.

The higher levels of soluble collagen in the chordae throughout gestation further reflect the differential development of the chordae versus the leaflet. Yet, while the levels were higher in the chordae, there was no change in these concentrations over gestation in either tissue. Further, levels of soluble collagen were surprisingly low in both tissues; this is unexpected in developing fetal collagenous tissues which require collagen deposition for their formation. Procollagen molecules are synthesized within the rough endoplasmic reticulum before being released into the cytosol where they self-assemble into trimers, forming the collagen molecule. After excretion from the cell and post-translational modifications, soluble collagen molecules are deposited and crosslinked by the enzyme family of lysyl oxidases, forming insoluble collagen fibrils. Many of these crosslinked fibrils bundle together to form larger collagen fibers [65]. The accumulation of insoluble collagen in the fetal chordae and leaflet, with unchanging concentrations of soluble collagen, suggests an elevated rate of collagen crosslinking during gestation, possibly via an upregulation of lysyl oxidases. The faster accumulation of mature collagen in the chordae may correspond to increased lysyl oxidase activity in comparison to that in the leaflet.

Similar patterns during gestation were seen in collagen crimp wavelength, with its development occurring at a faster rate in the chordae versus the leaflet. While the exact mechanism of crimp formation remains unknown [66], it has been suggested that cellular contraction triggered via mechanotransduction leads to the formation of crimp [67], with crimp wavelength increasing as tissues grow and elongate in the fiber direction [66,68,69]. While collagen crimp developed at the same rates throughout gestation in the bovine leaflet and chordae, we saw consistently longer wavelengths in the chordae. One explanation is that collagen crimp formation and lengthening begin earlier in the chordae (or at least earlier than the interval examined). This aligns with our previous observations above, which suggest that the chordae are leading the leaflet in developmental maturation and achieve functional maturity (in terms of tensile loading bearing) earlier. 

There are interesting parallels between the developing fetal mitral valve and the simultaneous remodeling of the maternal mitral valve during pregnancy; both show an increase over gestation in leaflet area (predominately in the circumferential direction), number of chordae attachments, and lack of change in normalized strut chordal distance, as well as position. In terms of collagen structure, both the maternal and fetal leaflets demonstrate an increase in crimp wavelength over gestation [50,59]. Similarities are also observed in collagen remodeling in both fetal and maternal valves: a rapid accumulation of insoluble crosslinked collagen with extremely low concentrations of newly-synthesized soluble collagen [50]. This similarity suggests that an upregulation of lysyl oxidase activity may be an important mechanism in both maternal valve remodeling and fetal valve development. Further work on fetal and maternal valves should examine lysyl oxidase activity. High-performance liquid chromatography techniques also would provide direct analysis of the changing crosslink types over gestation.

While the chordae led the leaflet in collagen deposition, maturation, and crimp formation, the leaflet led the chordae in terms of elastic fiber development. Elastic fibers first appeared near the fixed edge of the leaflet early in the second trimester, then accumulated into the rest of the leaflet over the remaining gestation period. By contrast, elastic fibers did not appear in the chordae until the third trimester. Thereafter, chordae elastic fibers rapidly accumulated at the outer layers, reaching an adult-like appearance [58,61] by full term. The formation of this elastic fiber “jacket” may signal the end of chordae multiplication, as the number of chordae becomes fixed after birth. This is sensible as the elastin gene is turned off at maturity [70,71] and if chordae were to continue dividing into adulthood, an elastin jacket could not be synthesized to arrest chordae division. While this study focused on the timing of the presence of elastic fibers, future work should include histological analysis of the leaflet in cross-section to better capture changes in elastic fiber organization over gestation. Further, immunohistochemistry for the specific elastic fiber components (fibrillins, tropoelastins) in earlier gestation fetal calves would better elucidate the time course of elastic fiber assembly in the developing mitral valves.

The observed differential development of the bovine fetal mitral valve leaflet and chordae tendineae supports previous work in chick and mice models that have suggested compartmentalized cell signaling during development in these tissues [30,72,73]. Distinct populations of cells in the developing endocardial cushions [71] direct a tendon-like development in the chordae versus a more cartilaginous-like development in the leaflet [30]. Specifically, leaflet-forming cells are responsive to bone morphogenetic protein 2 (BMP2) that induces expression of the cartilage markers sox9 and aggrecan, whereas chordae-forming cells are responsive to fibroblast growth factor 4 (FGF4) that induces the expression of tendon markers scleraxis and tenascin [72]. Interestingly, other FGF proteins have been shown to downregulate elastin expression [74,75], raising the possibility that FGF expression in the chordae may be suppressing elastic fiber synthesis in early development. The appearance of elastic fibers during the third trimester in the chordae may be triggered by increasing glucocorticoids [76], as they increase elastin gene expression [77,78] and may override the suppressive signal from FGF. Immunohybridization studies conducted in the bovine model are needed to determine if tendon-associated markers, such as FGF, are present in the developing bovine chordae. 

## 5. Conclusions

In summary, this study demonstrated differential development of the mitral valve anterior leaflet and chordae tendineae in a bovine model—differing from that in small mammals with shorter gestational periods, and strikingly similar to that observed in humans. Elucidating the mechanisms of mitral valve development over a longer gestation (like humans) could aid in identifying important congenital defects and other underlying causes of adult mitral valve disease. Finally, as tissue engineering strategies shift towards adopting a more developmentally-focused approach, these findings can inform the design criteria for heart valve tissue engineering. 

## Data Availability

All datasets will be made publicly available at the time of publication and are accessible by request of the corresponding author.
